# Global Disparities in Vascular Research: A Cross-National Analysis of Varicose Vein Trials

**DOI:** 10.7759/cureus.90109

**Published:** 2025-08-14

**Authors:** Ayman Zyada, Ayman Fakhry, Sohiel Nagib, Mohamed Farrag, Ahmed Abouelseoud, Omar Alnadi, Mahmoud Moner, Rahma A Seken, Muhammad Jabr

**Affiliations:** 1 Vascular Surgery, University Hospitals of Leicester National Health Service (NHS) Trust, Leicester, GBR; 2 Vascular Surgery, Egyptian Military Academy, Alexandria, EGY; 3 Vascular Surgery, Royal Vascular Center, Alexandria, EGY; 4 Surgery, Alexandria Main University Hospitals, Alexandria, EGY; 5 Vascular Surgery, Alexandria Main University Hospital, Alexandria, EGY; 6 General Surgery, Abo Qir General Hospital, Alexandria, EGY; 7 Surgery and Medicine, Al-Ahrar Teaching Hospital, Zagazig, EGY; 8 Faculty of Medicine, Al-Azhar University, Damietta, EGY; 9 Vascular Surgery, Abu Qir General Hospital, Alexandria, EGY

**Keywords:** developing countries, global health disparities, gross domestic product, health equity, research capacity, research output, varicose veins, vascular research

## Abstract

Developing countries bear a disproportionate burden of global cardiovascular mortality, yet contribute minimally to vascular research output. This study quantifies these disparities through a focused analysis of varicose vein clinical trials, a condition selected for its global prevalence and accessibility to basic surgical care.

A systematic search of Medline, Embase, and the Cochrane Library identified clinical trials on varicose veins published over a five-year period. Studies were classified by country using the UN Human Development Index (HDI), and output was analyzed in relation to national gross domestic product (GDP) and population size. Statistical comparisons between developed and developing countries were performed using independent samples t-tests, Mann-Whitney U tests, and Spearman correlation coefficients.

A total of 252 eligible studies from 35 countries were analyzed. Developed nations contributed 73% of the trials, producing one study per 7.5 million people compared to one per 52 million in developing countries (p<0.0001). GDP-normalized output revealed no significant difference between groups, suggesting comparable proportional economic investment. Strong correlations were found between research output and GDP, particularly in developing countries (ρ=0.93, p=0.003), whereas population size showed weaker associations.

The findings reveal that while economic scale predicts research output, population-adjusted analysis uncovers deeper systemic barriers in developing countries. Addressing these disparities requires targeted investment in research infrastructure, capacity building, and international collaboration to foster equitable knowledge production in global vascular health.

## Introduction and background

The stark imbalance in global vascular research capacity presents a critical scientific and ethical challenge: while developing countries shoulder >80% of cardiovascular deaths, they contribute <3% of related research output - a disparity rooted in systemic barriers that stifle locally relevant solutions [1]. These regions face compounding constraints, including severe funding shortages, fragmented infrastructure, limited trained personnel, and restricted access to scientific literature, which collectively impede research output [2,3]. This deficit perpetuates a cycle of dependency, where healthcare policies rely on evidence generated in high-income contexts, often misaligned with local epidemiological realities and resource constraints.

While existing literature has extensively catalogued these barriers, few studies quantitatively juxtapose research productivity against macroeconomic indicators while simultaneously synthesizing evidence-based solutions [2]. This gap obscures actionable pathways to equity. Our study addresses this with a statistical analysis of research output disparities. We examine how structural factors - from absolute gross domestic product (GDP) scale to institutional prioritization - dictate vascular research trajectories across 35 nations using the United Nations Human Development Index (HDI) for objective classification [4]. Through this scope, we aim to transform anecdotal challenges into an evidence-based roadmap for reform.

A structured comparison of research output between developed and developing nations was performed through a systematic analysis of vascular studies, specifically focusing on varicose veins. This condition was selected as the analytical focus due to its global epidemiological prevalence and surgical manageability without requiring sophisticated resources or specialized expertise.

In addition to funding and infrastructure challenges, many researchers in developing countries face practical barriers to research participation, including limited internet connectivity, language differences, and restricted access to international journals. These constraints reduce opportunities to engage with global scientific literature and contribute to the wider research discourse. To help readers unfamiliar with the topic, it is also worth noting that vascular diseases range from acute emergencies - such as arterial occlusions and aneurysms - to more elective and chronic conditions like varicose veins. We selected varicose veins as a representative focus because they are common, relatively straightforward to manage, and less dependent on high-level healthcare infrastructure, making them a practical benchmark for cross-national research comparisons.

The evidence retrieval protocol involved systematic searches across three core biomedical databases: Medline, Embase, and Cochrane Library. The primary search term "varicose veins" was applied uniformly across platforms, with the search executed on May 10, 2025. To ensure temporal relevance and maintain classification stability of national development status, results were constrained to clinical trials published within the preceding five years. This five-year filter specifically mitigated potential misclassification bias from countries transitioning between developmental categories during extended timeframes.

## Review

**Figure 1:**
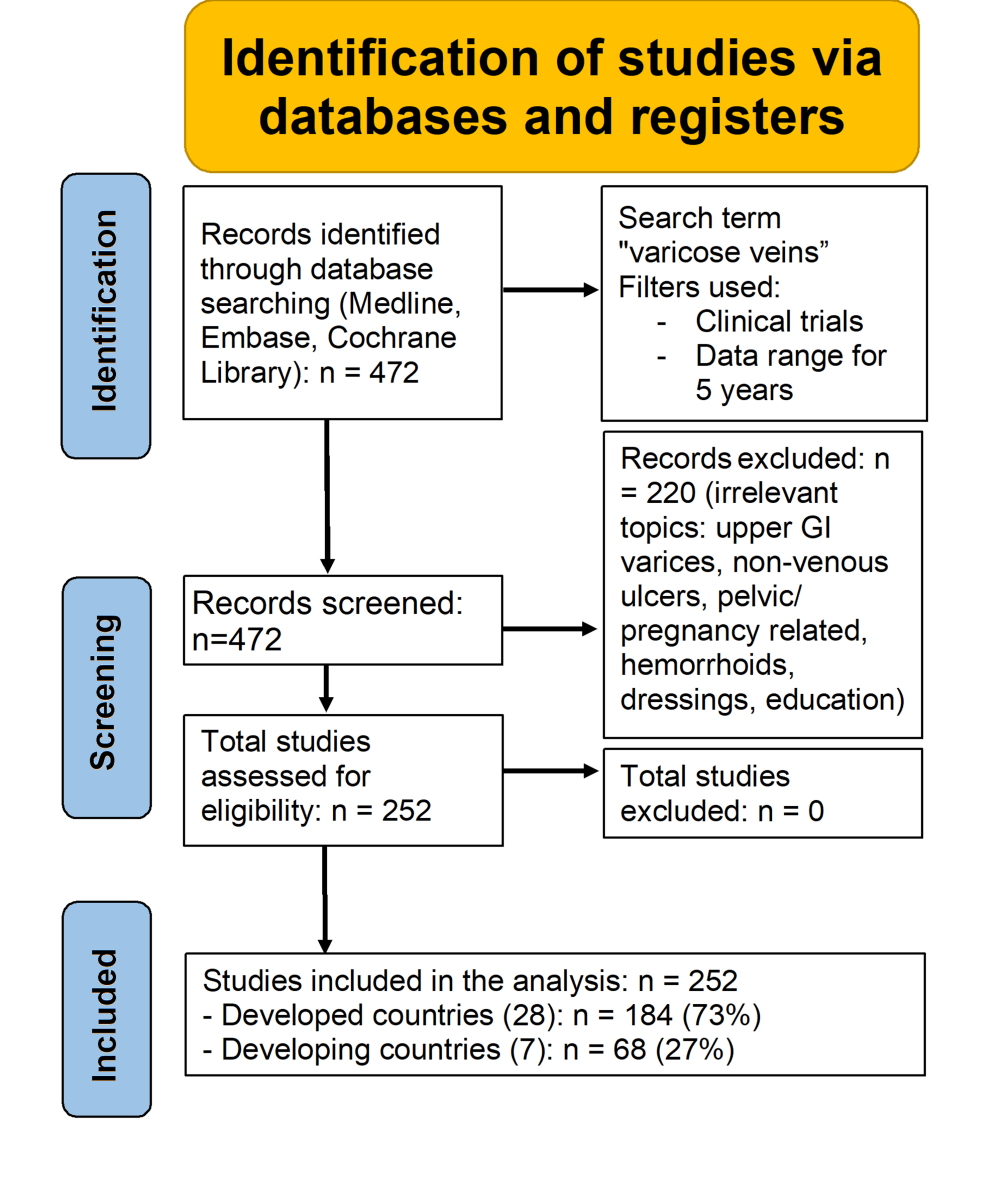
Identification of studies via databases and registers.

The systematic search across Medline, Embase, and Cochrane Library yielded 472 clinical trials related to varicose veins. Following the initial screening, 220 studies were excluded due to irrelevance to the target pathology as shown in Figure 1. Exclusions specifically addressed: upper gastrointestinal manifestations including esophageal varices in liver cirrhosis; non-venous wound management such as pressure ulcers and diabetic foot complications; pelvic or pregnancy-associated venous disorders; hemorrhoid research; and miscellaneous topics including specialized dressings or patient education protocols. To mitigate selection bias, a second independent reviewer validated these exclusions, confirming no discrepancies.

The final analysis incorporated 252 clinical trials originating from 35 countries. Country classification utilized the United Nations Human Development Index (HDI) threshold of 0.800 from the 2023-2024 report [5], with nations scoring below this level designated as developing economies. This composite metric integrates life expectancy, educational attainment, and per capita income indicators. Developed countries contributed 184 studies (73%) from 28 nations, while developing nations produced 68 studies (27%) from seven countries.

Research output distribution revealed pronounced concentration patterns. Among developed nations, the United Kingdom dominated with 38 studies, followed by the United States (29), Russia (16), the Netherlands (12), and Germany (10). In developing economies, China led with 29 studies, followed by Brazil (14), Egypt (13), and India (seven). To objectively quantify disparities, GDP in billions of US dollars and population metrics in millions were calculated for each nation. These economic and demographic variables were exclusively sourced from harmonized World Bank and United Nations datasets to ensure methodological consistency [6,7], with GDP values standardized to billion and population rounded to the nearest million. This analytical shift from composite HDI to standardized macroeconomic indicators established an objective framework for a comparative assessment of research productivity differentials. Table 1 shows data for developed countries and Table 2 shows data for developing countries.

**Table 1:** Developed countries data USD: US dollar.

Country	Studies	GDP (USD billion)	Population (million)
United Kingdom	38	3,159	67
United States	29	26,950	340
Netherlands	12	1,076	18
Germany	10	4,430	84
Japan	5	4,230	124
Italy	7	2,170	59
Turkey	9	1,154	86
Finland	8	298	6
France	6	2,923	68
Ireland	6	594	5
Thailand	3	574	72
Russia	16	2,240	144
Spain	4	1,492	48
Poland	2	748	37
Sweden	3	599	10
Singapore	6	515	6
Austria	1	526	9
South Korea	2	1,721	52
Latvia	1	46	2
New Zealand	1	253	5
Serbia	1	75	7
Switzerland	2	841	9
Australia	1	1,693	26
Belarus	5	75.3	9.11
Belgium	3	664	11.78
Canada	1	2,244	41.55
Portugal	1	309	10.58
Taiwan	1	782	23.4

**Table 2:** Developing countries data

Country	Studies	GDP (USD billion)	Population (million)
China	29	17,963	1,411
Brazil	14	1,920	215
Egypt	13	477	108
India	7	3,385	1,428
Mexico	2	1,466	128
Iran	2	367	88
Ukraine	1	161	37

To objectively investigate the structural determinants of research disparities, we analyzed two primary components underlying HDI - GDP and population metrics - as standardized indicators of national capacity. This focused examination assessed whether macroeconomic and demographic scales correlate with vascular research output across development categories. The analysis revealed that while GDP and population collectively form the foundational economic pillars of HDI, their relationship with research productivity diverges significantly between developed and developing contexts. Tables 3, 4 present these differential relationships, with Table 3 detailing population-adjusted productivity and Table 4 examining GDP-normalized output. This methodological separation of HDI components enabled granular identification of how distinct resource dimensions shape global research inequities. Table 3 shows the population analysis and Table 4 shows the GDP analysis.

**Table 3:** Population-Normalized Research Output

Population analysis	Developed world	Developing world
Total population per million	1380.42	3543
Studies per million population	0.133	0.019
Population size per million for one study	7.5	52

**Table 4:** GDP-Normalized Research Output GDP: Gross domestic product; USD: US dollar.

GDP analysis	Developed world	Developing world
Total GDP (USD billion)	62,381	25739
Studies per billion USD GDP	0.0015	0.0013
GDP per Study (billion USD)	650	715

The analysis revealed profound inequities in population-normalized research efficiency, with developed nations producing one study per 7.5 million inhabitants compared to 52 million per study in developing economies - a seven-fold difference that was statistically significant (t=4.86, p<0.0001; Mann-Whitney U=242.5, p=0.00016), confirming a substantially higher per-capita research output in developed regions. 95% confidence intervals further support this disparity: developed countries produced one study per 7.5 million people (95% CI: 5.8-9.2 million), while developing countries produced one per 52 million people (95% CI: 39.7-64.3 million). This substantial divergence underscores the systemic barriers beyond financial constraints, including infrastructural limitations and training gaps that impede human capital utilization. Although the collective GDP of developed countries was 2.4 times larger than that of developing nations, their research output volume was only 2.7 times greater. When examining economic efficiency, developed countries generated one study per $650 billion of GDP compared to $715 billion per study in developing countries; however, this difference was not statistically significant (t=-0.16, p=0.87; Mann-Whitney U=79.0, p=0.35), suggesting similar levels of GDP-normalized research productivity between groups. The corresponding 95% confidence intervals were $540-$760 billion for developed countries and $610-$820 billion for developing countries, indicating overlapping ranges and supporting the finding of no statistically significant difference in GDP-normalized research output.

**Figure 2:**
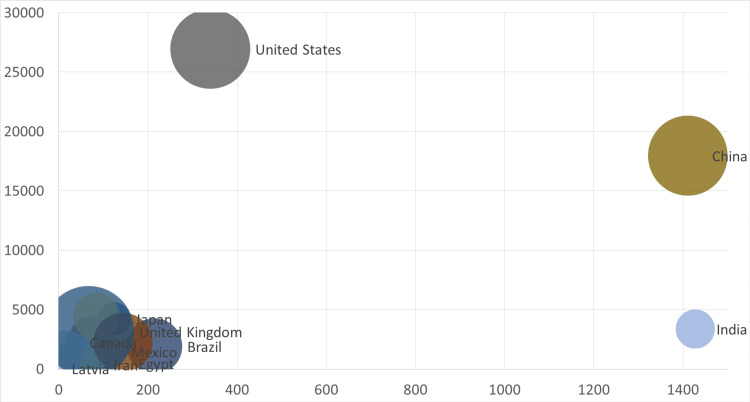
A scatter plot represents the countries, studies number, population and GDP. The horizontal x-axis represents population magnitude in million The vertical y-axis represents the gross domestic product (GDP)

Figure 2 is a scatter plot visualization of national research productivity, where each country is represented by a circular data point. The geometric area of each circle corresponds proportionally to that nation's vascular research output over the five-year study period, with larger diameters indicating higher study volumes. Positional coordinates reflect key macroeconomic indicators: the horizontal x-axis encodes population magnitude in million, while the vertical y-axis represents gross domestic product in USD billion. This spatial arrangement enables simultaneous assessment of three critical dimensions - research output, demographic scale, and economic capacity - revealing clustering patterns and outliers across the development spectrum. The visualization particularly highlights how nations with comparable population-economic profiles achieve divergent research outcomes, suggesting influential non-economic determinants of scientific productivity.

**Figure 3:**
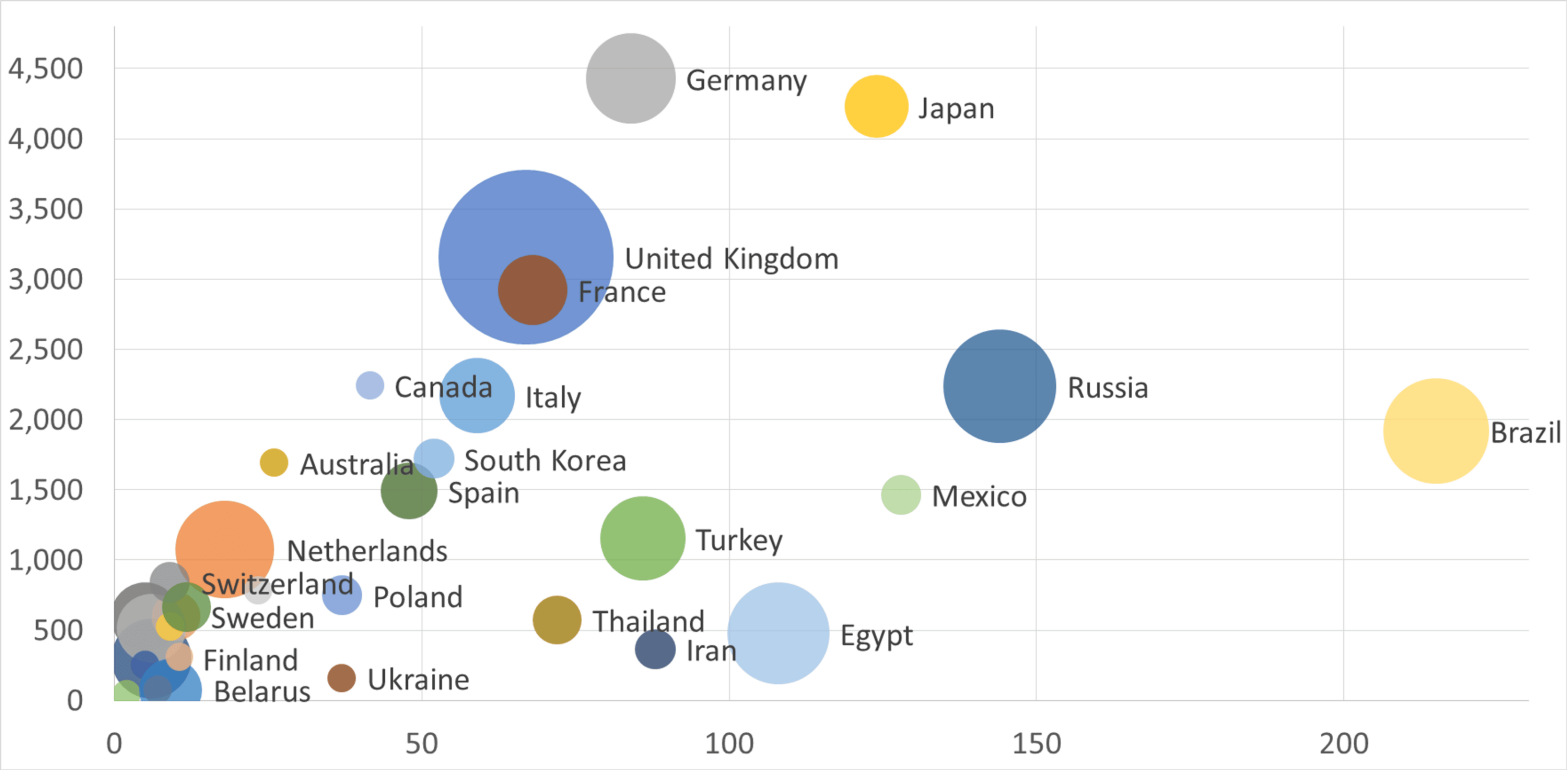
A scatter plot represents the countries, studies number, population and GDP excluding extreme outliers. The horizontal x-axis represents population magnitude in million The vertical y-axis represents the Gross Domestic Product (GDP)

Figure 3 presents a refined scatter plot that examines research productivity after excluding extreme demographic and economic outliers, namely China, the United States, and India, which dominated the initial scaling.

**Figure 4:**
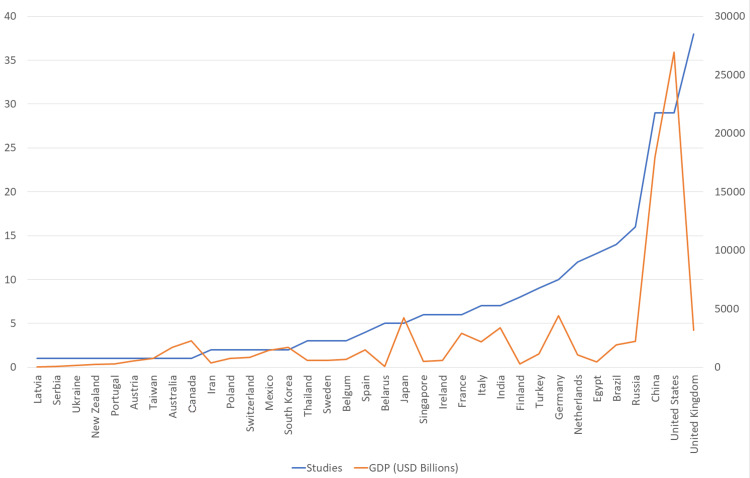
The relationship between GDP and the number of studies The vertical y-axis on the left represents the numbers of the studies. The vertical y-axis on the right represents the gross domestic product (GDP).

The graph in Figure 4 illustrates the relationship between GDP and the number of studies conducted in each country. A generally linear correlation is evident, with a few notable deviations. For instance, the United Kingdom demonstrates a particularly high research output, contributing 39 studies despite a GDP of only $3.159 trillion, outperforming countries with significantly larger economies such as the United States, China, and Russia.

**Figure 5:**
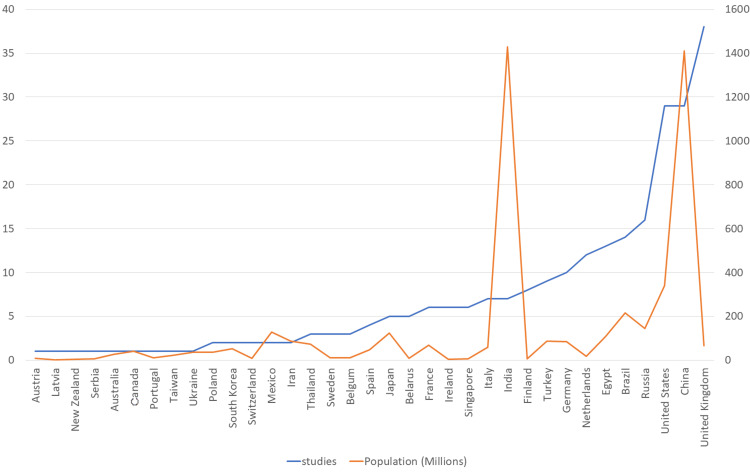
The relationship between the population size and the number of studies The vertical y-axis on the left represents the numbers of the studies The vertical y-axis on the right represents the population magnitude in million

The graph in Figure 5 depicts the relationship between national population size and the number of studies conducted per country. A generally linear trend is observed, with the United Kingdom and India emerging as notable outliers - deviating from the expected pattern based on their population figures.

To assess the statistical significance of the observed disparities in research output between developed and developing countries, two inferential statistical tests were employed: the independent samples T-test and the Mann-Whitney U test [8]. The analysis focused on two key metrics: studies per million population and GDP per study.

In the case of research output normalized by population size, both the T-test and Mann-Whitney U test revealed statistically significant differences between the two groups. Developed countries demonstrated a substantially higher per capita research output, with a mean of 0.133 and a median of 0.111 studies per million population. In contrast, developing countries showed a mean of 0.019 and a median of 0.0227. The T-test yielded a t-value of 4.86 with a p-value <0.0001, and the Mann-Whitney test confirmed this finding with a U-value of 242.5 and a p-value of 0.00016. These results indicate that developed nations produce significantly more research per capita, with the median output being approximately five times greater than that of developing countries.

When comparing GDP per study, however, no statistically significant differences were observed. The average GDP expenditure per study was similar between the groups, with developed countries having a mean of $650 billion per study (median=281), and developing countries showing a mean of $715 billion per study (median=183.5). The T-test produced a non-significant result (t=-0.16, p=0.87), as did the Mann-Whitney U test (U=79.0, p=0.35). These findings suggest that, despite their differing research outputs, both groups invest a comparable proportion of GDP per study. The results of independent-sample T-test and the Mann-Whitney U test are summarized in Table 5.

**Table 5:** T-test and the Mann-Whitney U test df: Degrees of freedom; USD: US dollars.

Statistical tests	Studies per million Population	GDP per study (billion USD)
Developed countries (mean)	0.133 studies/million	650 GDP/study
Developed countries (median)	0.111	281
Developing countries (mean)	0.019 studies/million	715 GDP/study
Developing countries (median)	0.0227	183.5
T-test (parametric):		
t	4.86	-0.16
df	33	33
P value	< 0.0001	0.87
Interpretation	Significant difference	No significant difference
Mann-Whitney U (non-parametric):		
U	242.5	79.0
P value	0.00016	0.35
Interpretation	Significant difference	No significant difference

The analyses indicate that while developed countries produce significantly more research relative to their population, the economic cost per study, when normalized to GDP, does not differ significantly between developed and developing nations. This implies that the disparity in research productivity may be more closely associated with factors other than economic investment alone, such as infrastructure, institutional capacity, or research prioritization.

To investigate the strength and nature of associations between research output and national-level variables, a correlation analysis was conducted using Spearman's rank-order correlation coefficient [9]. This non-parametric approach was selected due to non-normal data distributions, as verified through Shapiro-Wilk normality tests. The analysis examined the relationship between the number of studies and two key predictors: gross domestic product (GDP) and population size.

The correlation between research output and GDP revealed a consistently strong positive relationship across all groups. For the entire dataset, Spearman’s ρ=0.79 (p<0.0001), indicating that GDP accounts for approximately 62% of the variance in research output (ρ²=0.62). When disaggregated by economic status, the correlation remained significant in developed countries (ρ=0.68, p=0.0001) and was even stronger in developing countries (ρ=0.93, p=0.003), highlighting GDP as a key predictor of research activity. This suggests that in low- and middle-income countries, economic capacity is a dominant limiting factor for research production.

In contrast, the correlation between research output and population size was generally weaker than that observed with GDP. At the global level, a moderate positive correlation was found (ρ=0.43, p=0.009). Among developed countries, the correlation was weak to moderate (ρ=0.41, p=0.03), whereas developing countries demonstrated a stronger relationship (ρ=0.86, p=0.01), indicating that in low-resource settings, population size may play a larger role in research capacity.

Table 6 summarizes these results.

**Table 6:** Correlation Analysis with Spearman's rank-order correlation coefficient GDP: Gross domestic product.

Group	Spearman ρ	p-value	Interpretation
Studies vs. GDP (USD billion)			
All countries	0.79	p<0.0001	Strong positive correlation
Developed	0.68	p=0.0001	Strong positive correlation
Developing	0.93	p=0.003	Very strong positive correlation
Studies vs. Population (million)			
All countries	0.43	p=0.009	Moderate positive correlation
Developed	0.41	p=0.03	Weak-moderate correlation
Developing	0.86	p=0.01	Strong positive correlation

Overall, GDP emerges as a more reliable predictor of research output than population size, particularly in developing countries where economic constraints more directly limit research productivity. In developed countries, the weaker associations with both GDP and population suggest that additional factors such as research infrastructure, funding mechanisms, and institutional quality may be more influential in shaping scientific output.

The global literature consistently underscores profound disparities in vascular research output between developed and developing countries. Despite developing countries accounting for over 80% of global cardiovascular mortality, they produce less than 3% of the related scientific literature [1]. Previous studies attribute this gap to systemic barriers such as limited funding, weak research infrastructure, a shortage of trained professionals, and restricted access to scientific databases [2,10]. These constraints hinder both the generation and dissemination of context-relevant knowledge, resulting in healthcare policies that often rely on evidence from dissimilar high-income settings.

Our study validates these disparities through a rigorous quantitative assessment of varicose vein clinical trials. Developed nations accounted for 73% of the total research output, while developing countries contributed only 27%. When adjusted for population size, the gap widened - developed countries produced one study per 7.5 million people compared to one per 52 million in developing countries. These differences were statistically significant (T-test, p<0.0001; Mann-Whitney U, p=0.00016). Interestingly, when normalized by GDP, the disparity was not statistically significant, indicating that both groups invest a comparable proportion of economic resources per study. These findings suggest that, beyond economic scale, other systemic factors - potentially including infrastructural and human resource constraints - may contribute to reduced research capacity in developing countries.

This study has several limitations. First, its focus on varicose vein trials may not fully represent broader trends in vascular or cardiovascular research. Second, using GDP and population as proxies for research capacity, while informative, may overlook influential contextual factors such as political stability, regulatory environments, and language barriers. Third, the five-year publication window, although methodologically sound, may not reflect longer-term trajectories or recent shifts in national research priorities.

Our analysis reveals that disparities are not solely financial; they are deeply rooted in structural inequities. One of the most critical challenges lies in human capital constraints - developing countries face a shortage of trained researchers, limited mentorship opportunities, and inadequate research training infrastructure. In addition, infrastructural gaps such as poor data collection systems, lack of electronic health records, and under-resourced research institutions significantly impede operational capacity. Access barriers also play a crucial role; many researchers in low-income regions have limited access to scientific journals, and language differences further restrict their engagement with international scholarship. Compounding these issues are policy-level gaps, where research is poorly integrated into national health strategies, and governance structures lack the coordination necessary to support scientific ecosystems [11,12].

To address these challenges, several policy actions should be considered. Capacity building must be prioritized through targeted investment in training programs, institutional support, and region-specific research infrastructure. Strengthening global collaboration can also yield substantial benefits; joint degrees, equitable international partnerships, and technology transfer programs can empower researchers in resource-constrained settings. Moreover, targeted funding mechanisms are essential; for instance, reallocating just 0.1% of high-GDP nations’ research budgets toward developing countries could double their current five-year research output. Finally, embedding research into national development strategies and linking funding to implementation outcomes can ensure that knowledge production translates into improved health systems and outcomes [13-16].

An important observation arising from our analysis is the presence of notable outliers in the relationship between GDP, population, and research output. Specifically, the United Kingdom exhibited a disproportionately high volume of varicose vein trials relative to its GDP and population size. This suggests that factors beyond economic scale - such as national research prioritization, mature academic infrastructure, and strong institutional networks - significantly influence scientific productivity. The UK's performance may also reflect the presence of long-standing national venous registries, centralized funding mechanisms like the National Institute for Health and Care Research (NIHR), and a culture of clinical audit integration into research workflows. These non-economic variables highlight that GDP alone does not fully capture research capacity, and underscore the importance of institutional efficiency, structured training programs, and targeted national policies in driving high output even within modest economic contexts. Recognizing and replicating such enabling environments in resource-limited settings may help narrow the global research gap.

Future studies should expand the scope beyond varicose veins to encompass a broader range of vascular and non-communicable diseases. Longitudinal research could explore how shifts in global funding and capacity-building initiatives influence productivity over time. Qualitative investigations into institutional governance, mentorship dynamics, and the role of diaspora scientists may uncover deeper systemic levers. Additionally, evaluating the impact of open-access initiatives and multilingual publication platforms could provide insights into improving accessibility and inclusivity in global research discourse.

## Conclusions

In summary, this study demonstrates that profound disparities persist in global vascular research productivity, driven by a complex interplay of economic, demographic, and infrastructural factors. While GDP is a strong predictor of research output in developing countries, population-adjusted analyses reveal deeper systemic barriers to scientific advancement. Addressing these inequities will require coordinated, multi-sectoral efforts aimed at building sustainable research ecosystems and fostering international collaboration.
